# Supplementary material for: Statistical Copolymers of N–Vinylpyrrolidone and 2–Chloroethyl Vinyl Ether via Radical RAFT Polymerization: Monomer Reactivity Ratios, Thermal Properties, and Kinetics of Thermal Decomposition of the Statistical Copolymers
Source: Polymers (Basel). 2023 Apr 21;15(8):1970. doi: 10.3390/polym15081970 (PMC10142009; doi:10.3390/polym15081970)
Supplement: Supplementary file 1 [file polymers-15-01970-s001.zip › polymers-2326478-supplementary.pdf]

Statistical Copolymers of *N*–Vinylpyrrolidone and 2–Chloroethyl Vinyl Ether via Radical RAFT Polymerization: Monomer Reactivity Ratios, Thermal Properties, and Kinetics of Thermal Decomposition of the Statistical Copolymers

Nikolaos V. Plachouras and Marinos Pitsikalis *

Industrial Chemistry Laboratory, Department of Chemistry, National and Kapodistrian University of Athens, Panepistimiopolis Zografou, 15771 Athens, Greece; plachouras.v.nikolaos@gmail.com
Correspondence: pitsikalis@chem.uoa.gr

Supporting Information

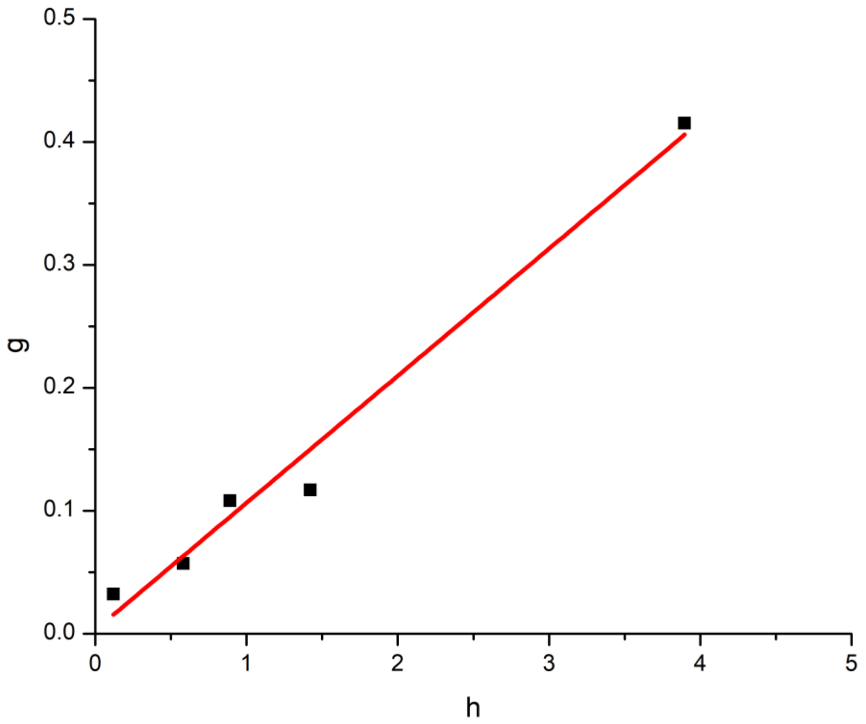

Figure S1. (F–R) plot of the P(NVP–stat–CEVE) copolymers.

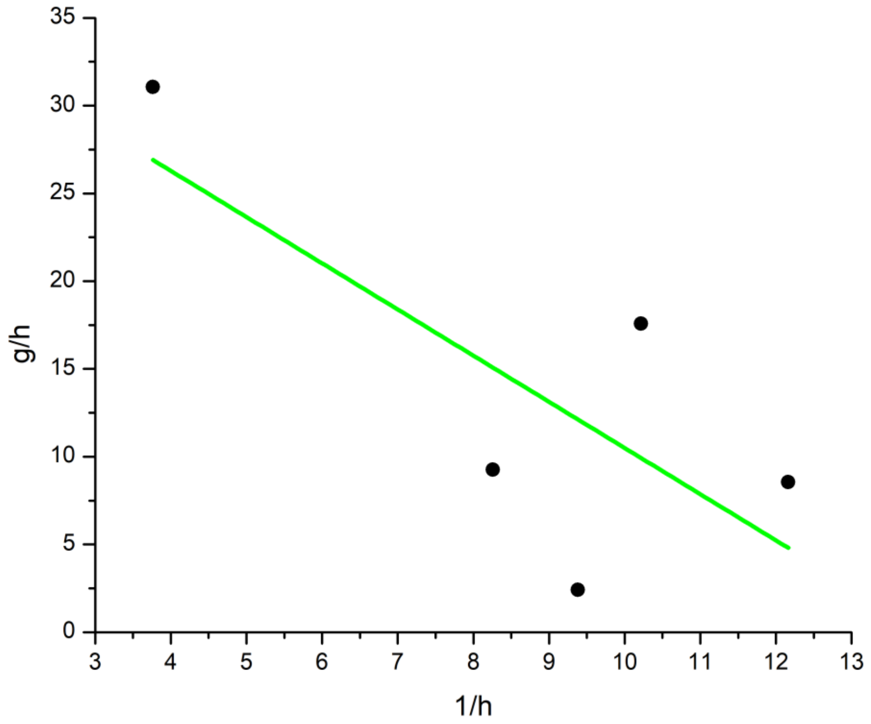

Figure S2. (inv. F–R) plot of the P(NVP–stat–CEVE) copolymers.

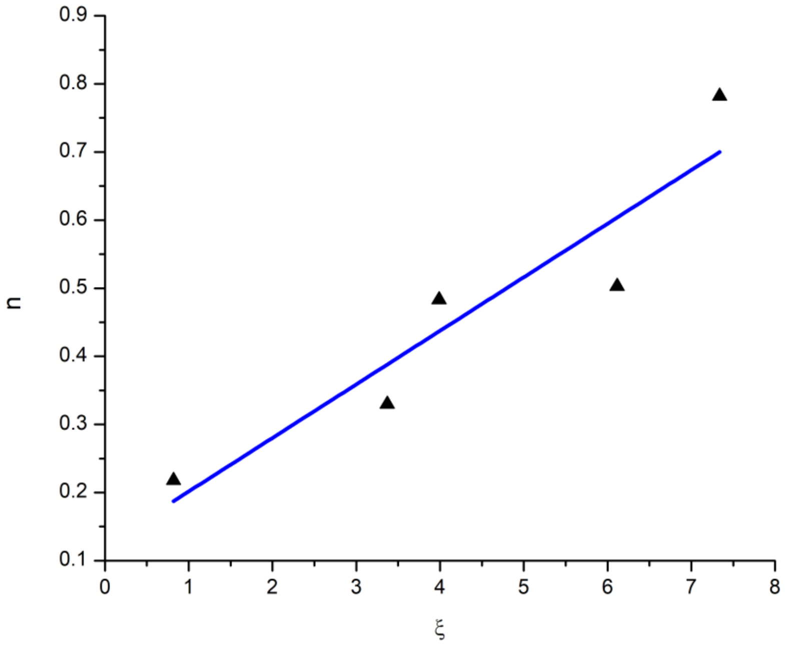

Figure S3. (K–T) plot of the P(NVP–stat–CEVE) copolymers.

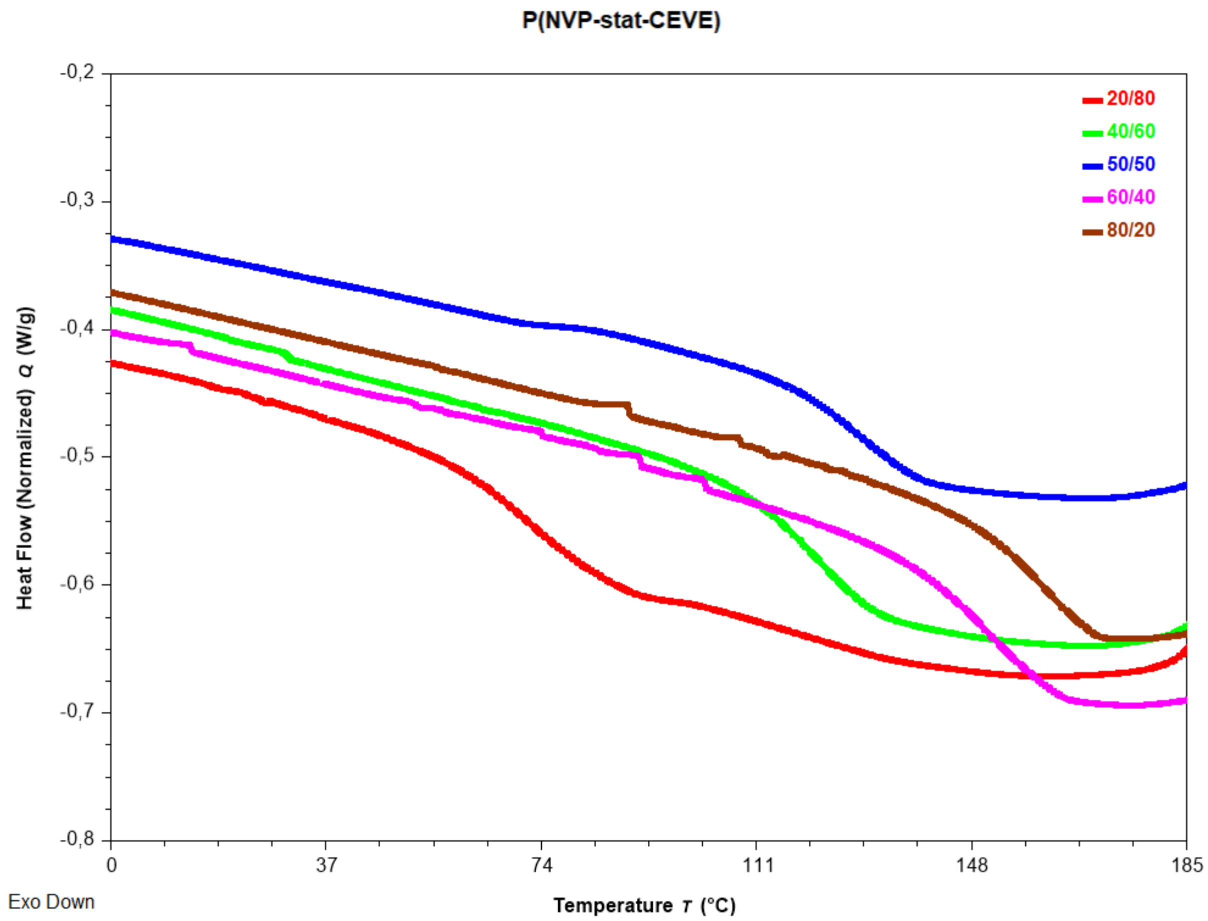

Figure S4. (DSC) thermograms for the P(NVP–stat–CEVE) copolymers.

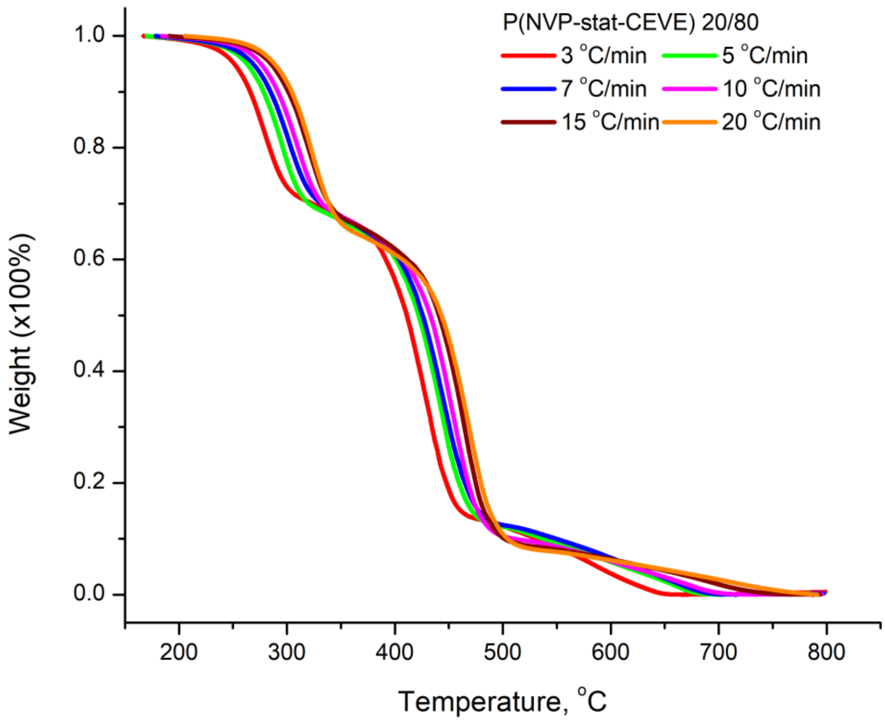

Figure S5. Weight loss with temperature under different heating rates for the P(NVP–stat–CEVE) 20/80.

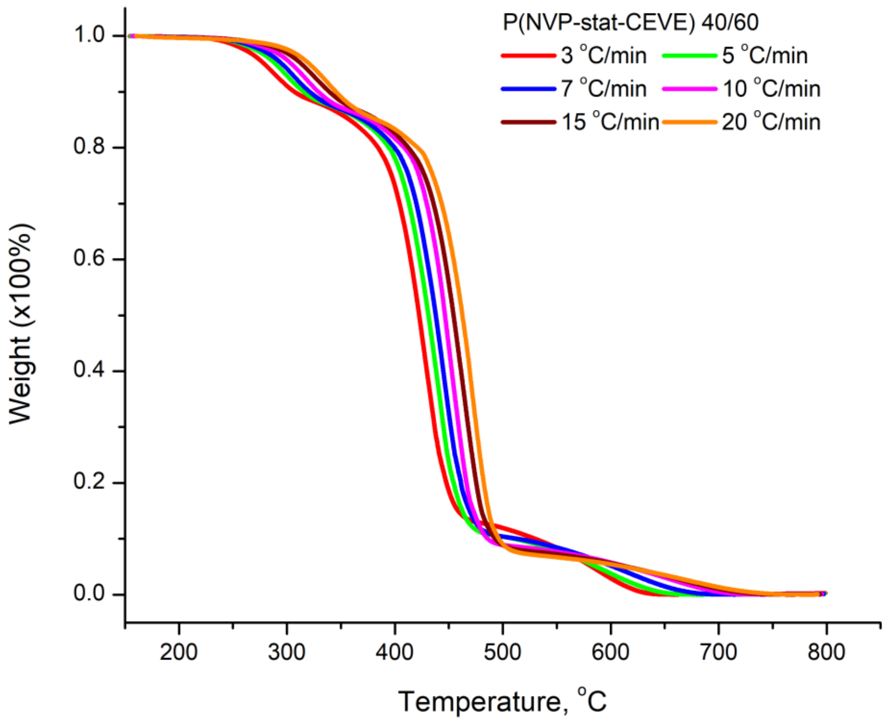

Figure S6. Weight loss with temperature under different heating rates for the P(NVP–stat–CEVE) 40/60.

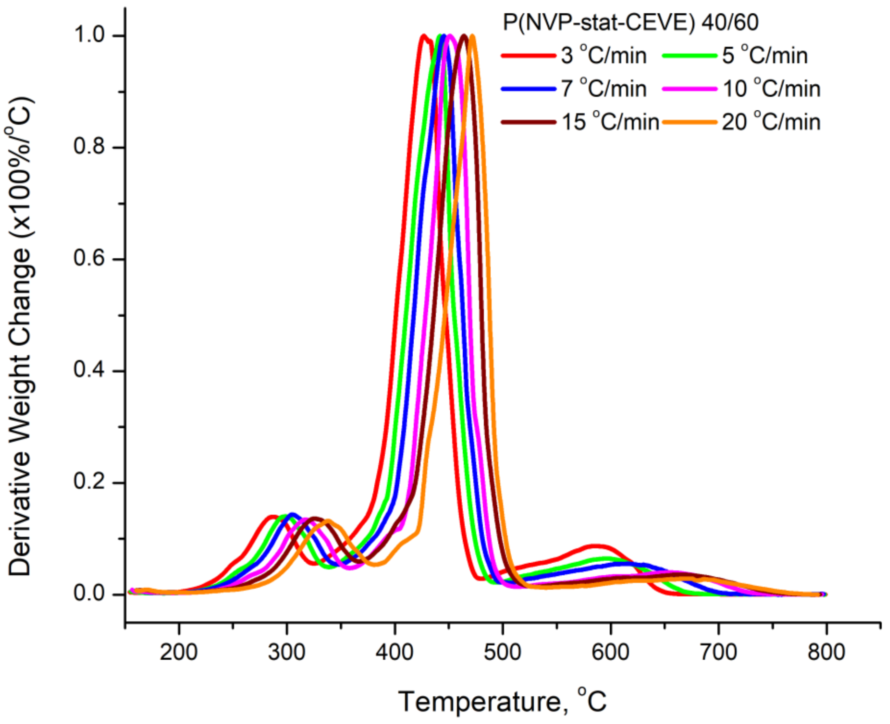

Figure S7. Derivative weight loss with temperature under different heating rates for the P(NVP–stat–CEVE) 40/60.

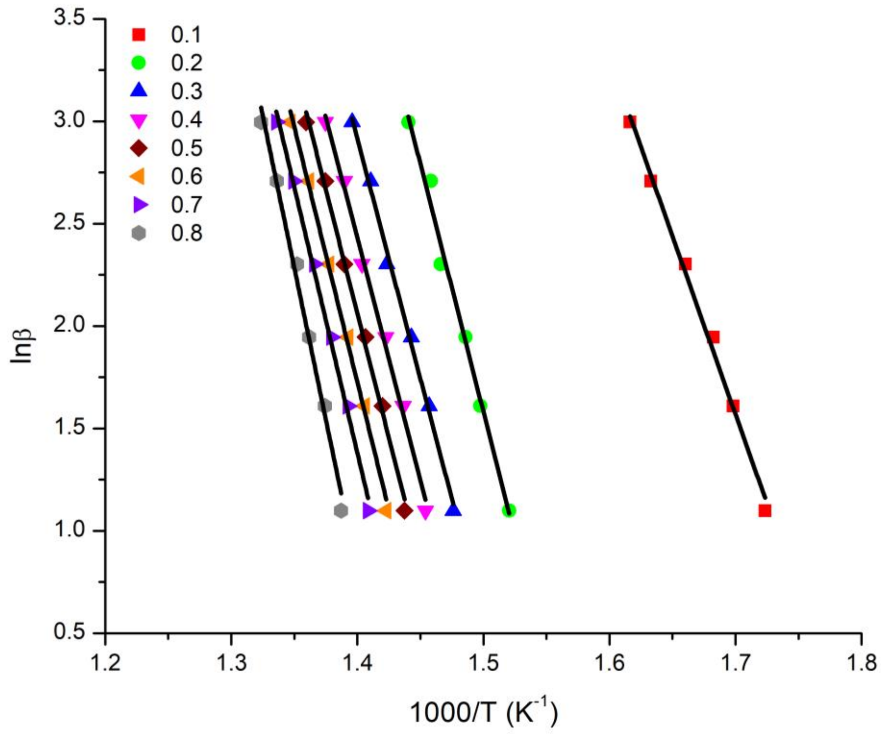

Figure S8. (OFW) plots for the sample P(NVP–stat–CEVE) 40/60.

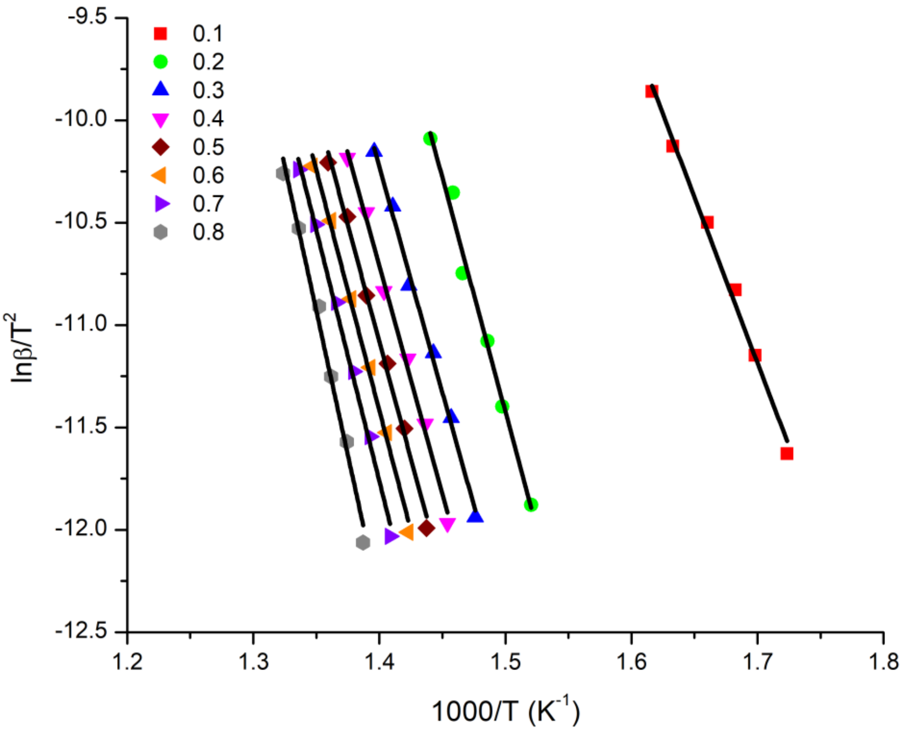

Figure S9. (KAS) plots for the sample P(NVP–stat–CEVE) 40/60.

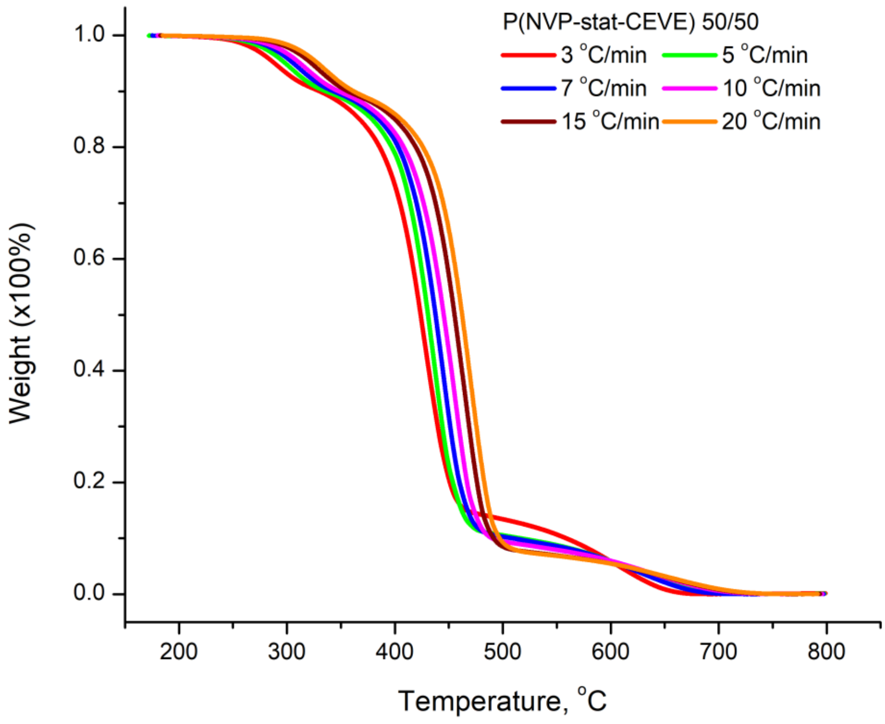

Figure S10. Weight loss with temperature under different heating rates for the P(NVP–stat–CEVE) 50/50.

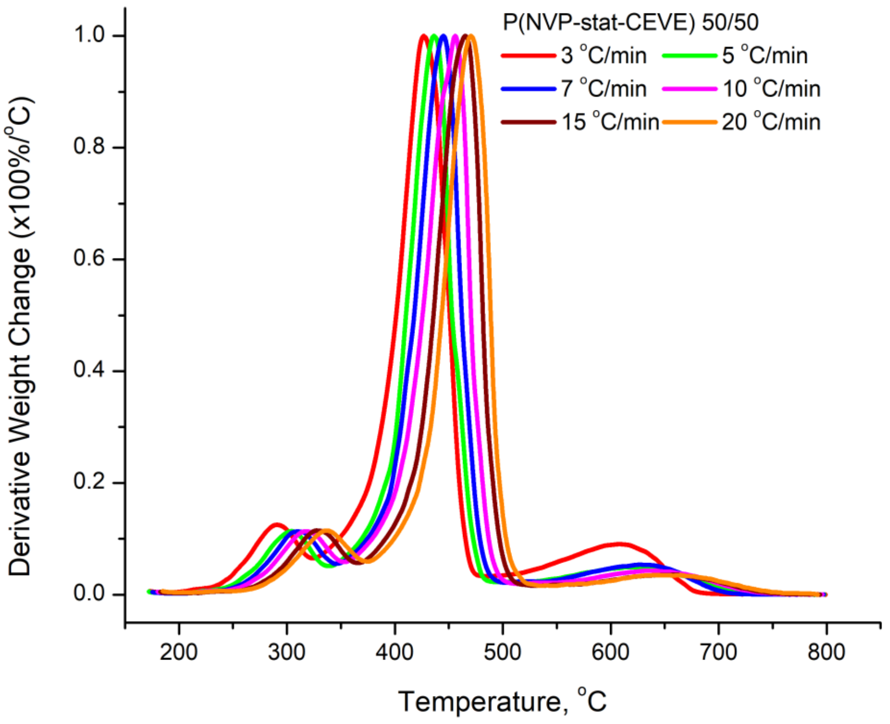

Figure S11. Derivative weight loss with temperature under different heating rates for the P(NVP–stat–CEVE) 50/50.

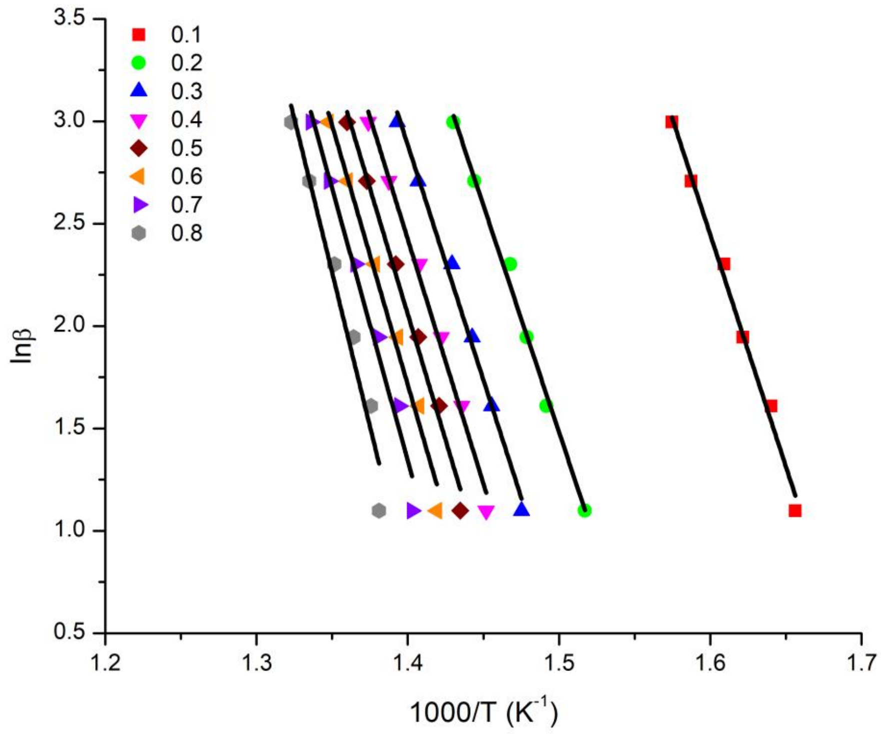

Figure S12. (OFW) plots for the sample P(NVP–stat–CEVE) 50/50.

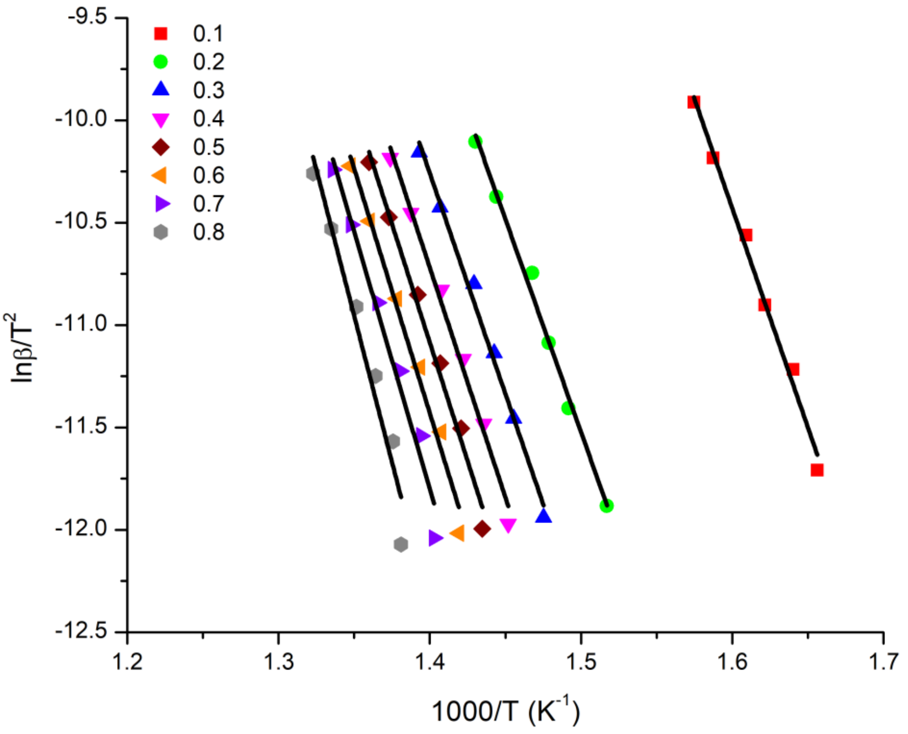

Figure S13. (KAS) plots for the sample P(NVP–stat–CEVE) 50/50.

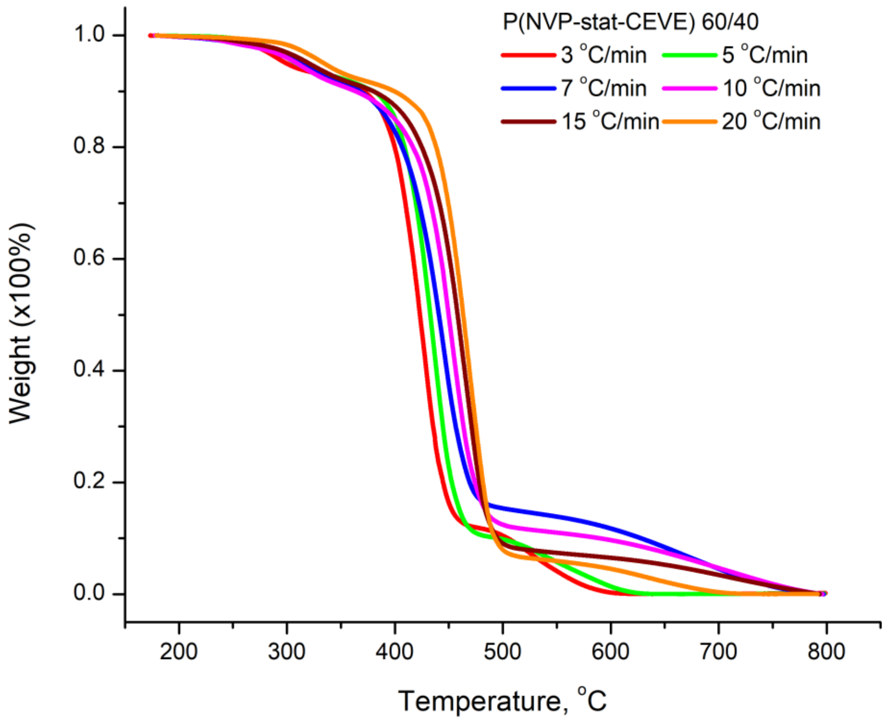

Figure S14. Weight loss with temperature under different heating rates for the P(NVP–stat–CEVE) 60/40.

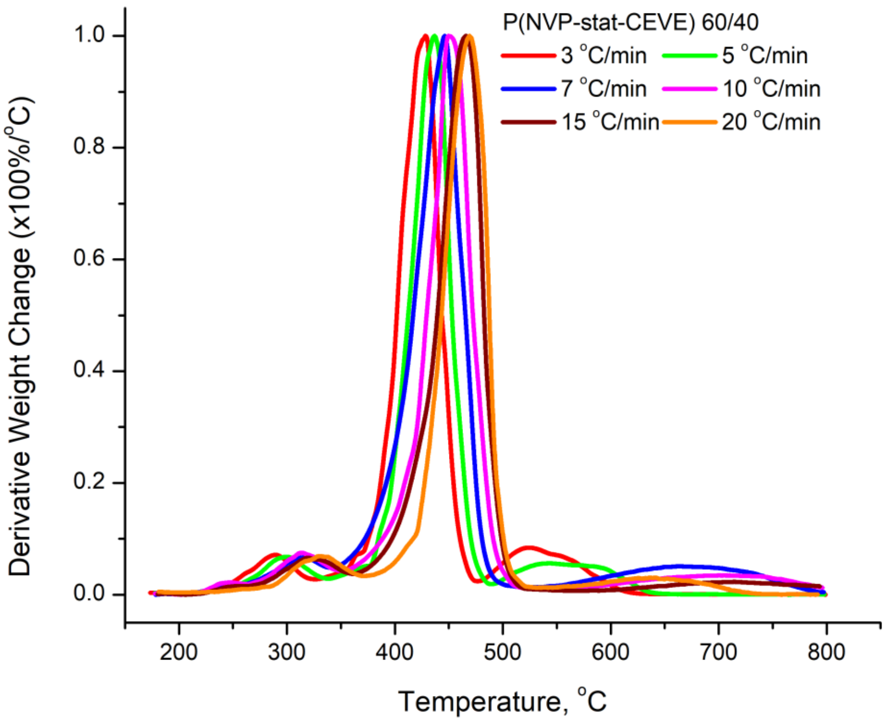

Figure S15. Derivative weight loss with temperature under different heating rates for the P(NVP–stat–CEVE) 60/40.

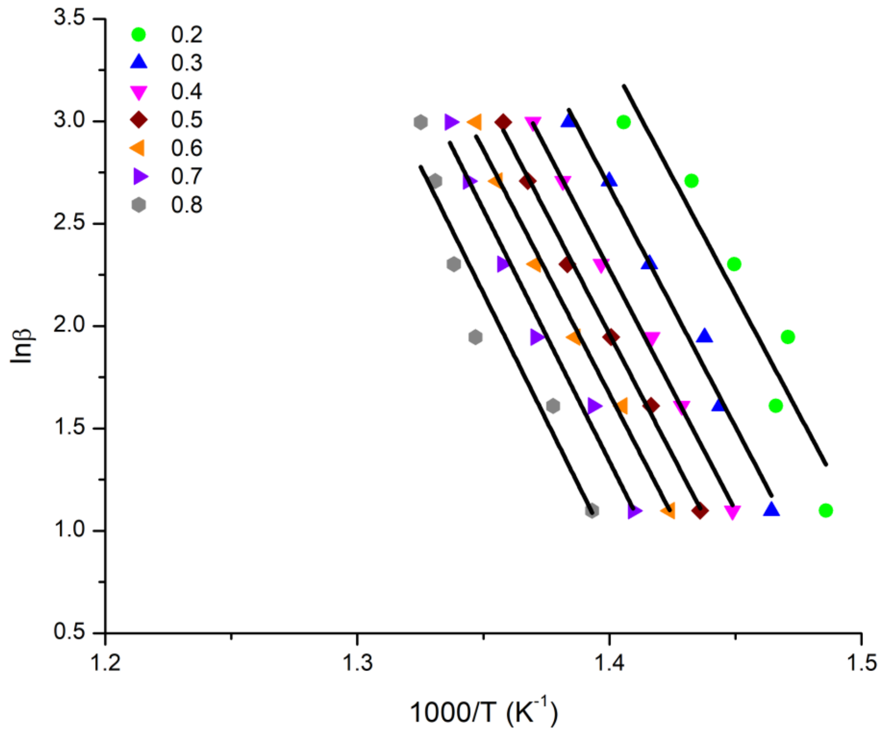

Figure S16. (OFW) plots for the sample P(NVP–stat–CEVE) 60/40.

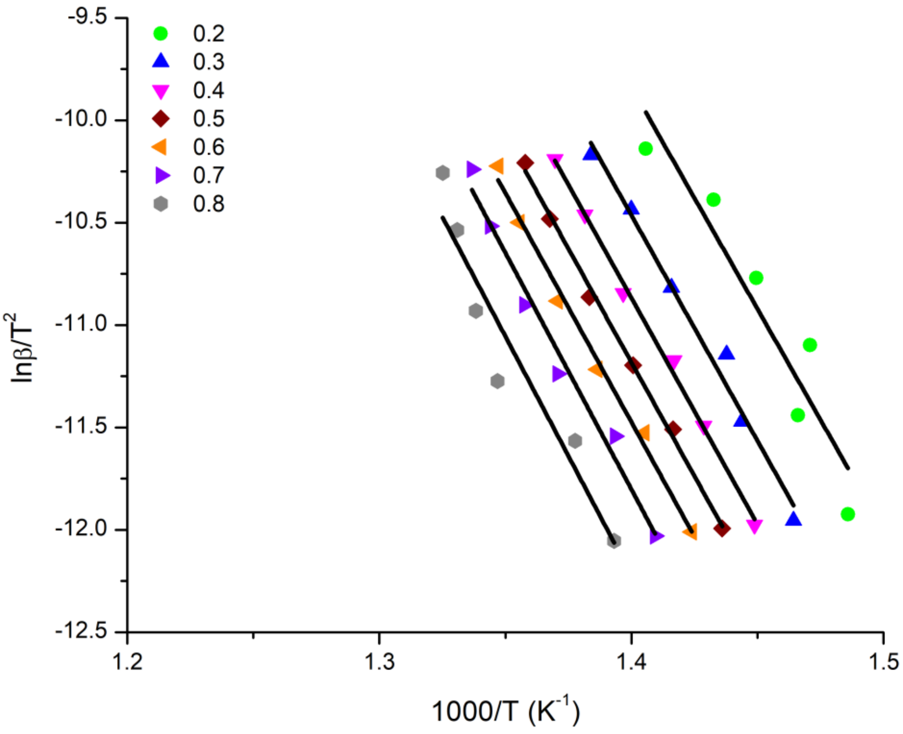

Figure S17. (KAS) plots for the sample P(NVP–stat–CEVE) 60/40.

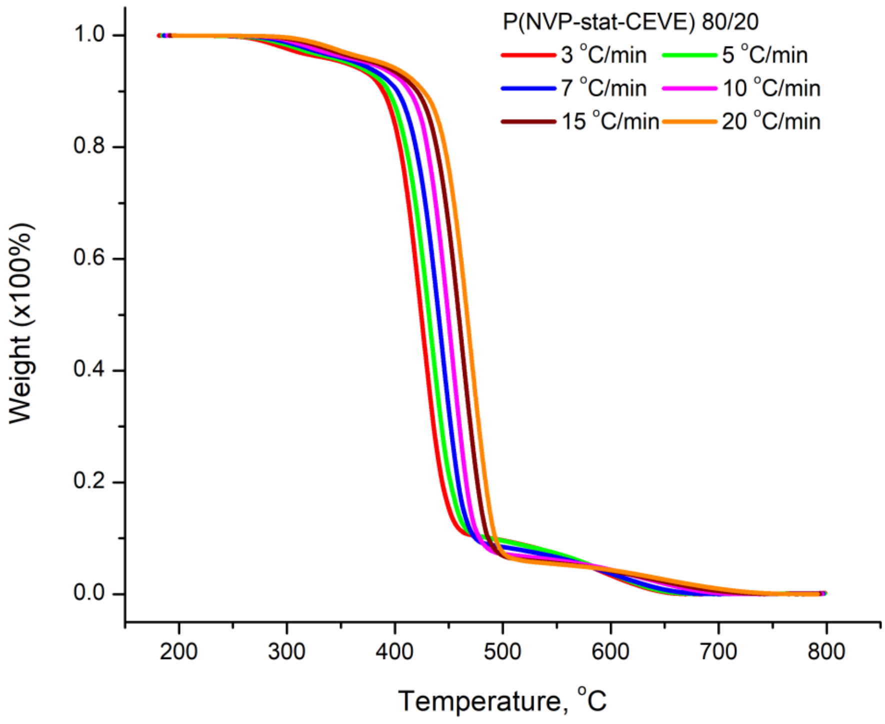

Figure S18. Weight loss with temperature under different heating rates for the P(NVP–stat–CEVE) 80/20.

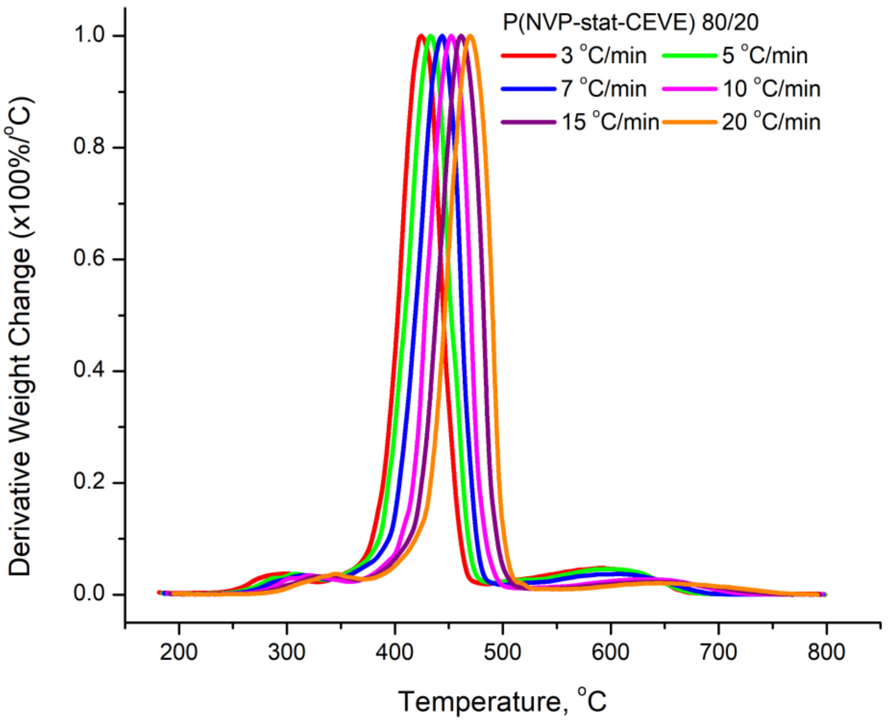

Figure S19. Derivative weight loss with temperature under different heating rates for the P(NVP–stat–CEVE) 80/20.

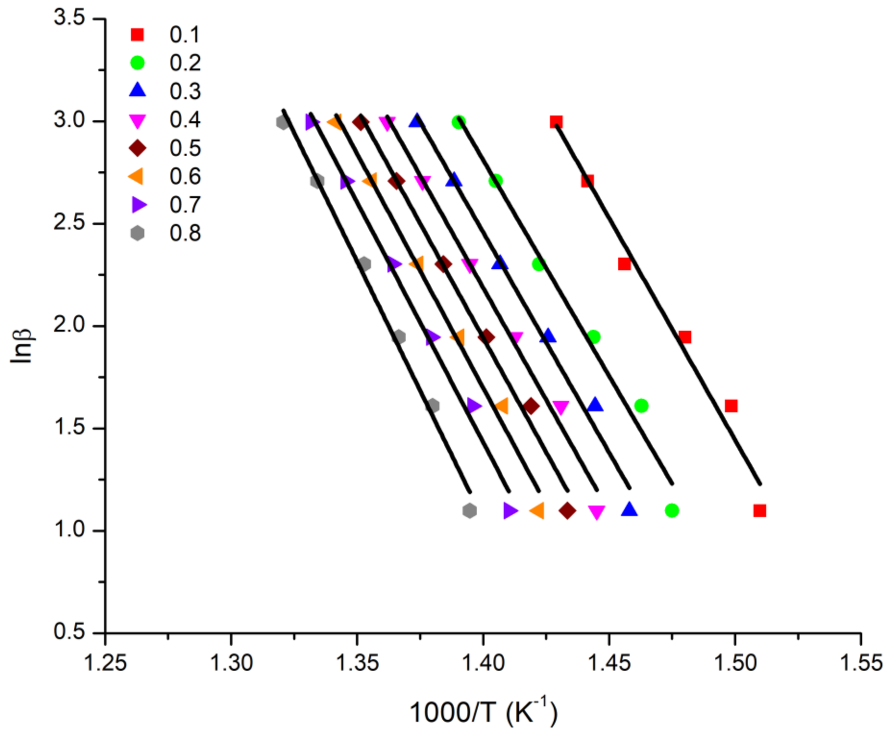

Figure S20. (OFW) plots for the sample P(NVP–stat–CEVE) 80/20.

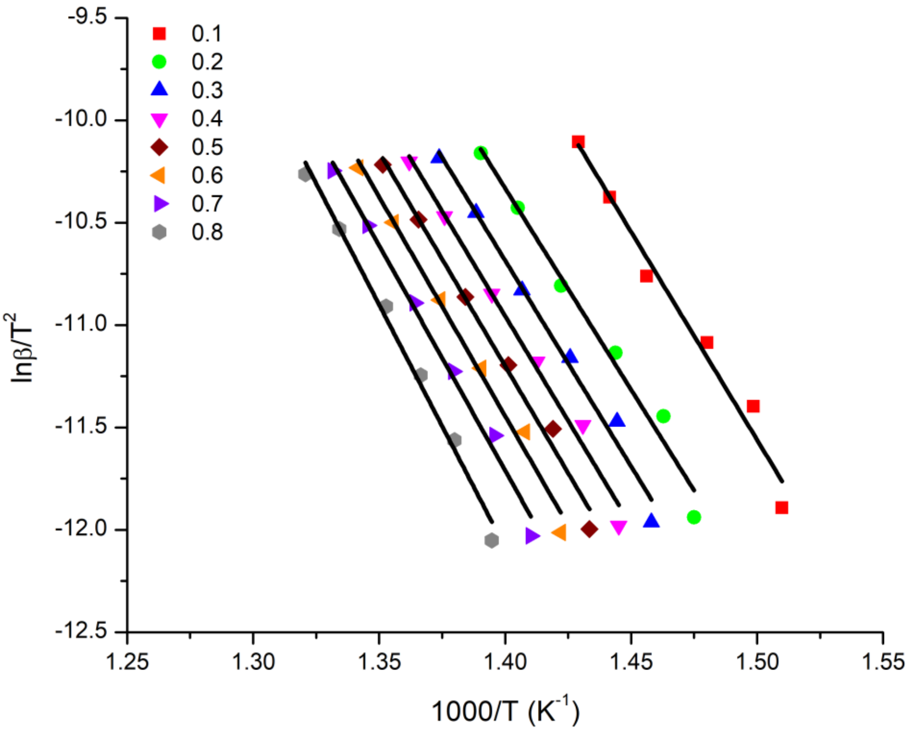

Figure S21. (KAS) plots for the sample P(NVP–stat–CEVE) 80/20.
